# PD205 “Medical Fund” – A Novel Approach To Granting Individuals Access To Cutting-Edge Therapies

**DOI:** 10.1017/S0266462324004276

**Published:** 2025-01-07

**Authors:** Michał Jachimowicz, Cezary Pruszko, Karolina Dziadek

## Abstract

**Introduction:**

Access to innovative and expensive medicines is a significant challenge for Poland’s healthcare system. These therapies often do not meet the reimbursement criterion that is currently set at three times the gross domestic product per capita. Nevertheless, there are ongoing efforts to identify funds that can cover the cost of innovative and expensive medicines.

**Methods:**

A new legal act established the Medical Fund, which is an addition to the regular National Health Fund. The Medical Fund finances medical technologies recognized by the Polish healthcare system as highly innovative or of high clinical value. Lists of such therapies are prepared by the health technology assessment agency, in consultation with clinical experts, and then approved by the Ministry of Health. Simplified marketing authorization applications based on a budget impact analysis, a more straightforward assessment process, and a separate budget may allow patients to access these therapies faster.

**Results:**

Since January 2022, ten highly innovative therapies have been funded for patients with conditions such as spinal muscular atrophy, acute hepatic porphyria, and primary hyperoxaluria. The reimbursement decisions were issued for a two-year period, during which data on treatment efficacy were collected. If the data collected after two years is insufficient to assess the treatment’s efficacy, the decision can be extended without an additional procedure for another two years. After two or four years, the marketing authorization holder must submit a reimbursement application based on a full health technology assessment report. Risk-sharing schemes based on clinical outcomes are mandatory.

**Conclusions:**

The Medical Fund has granted early access to modern therapies. The decision to continue funding for a particular drug depends on whether registry results confirm data from experimental studies.

